# Process evaluation of complex interventions: Medical Research Council guidance

**DOI:** 10.1136/bmj.h1258

**Published:** 2015-03-19

**Authors:** Graham F Moore, Suzanne Audrey, Mary Barker, Lyndal Bond, Chris Bonell, Wendy Hardeman, Laurence Moore, Alicia O’Cathain, Tannaze Tinati, Daniel Wight, Janis Baird

**Affiliations:** 1 DECIPHer UKCRC Public Health Research Centre of Excellence, School of Social Sciences, Cardiff University, Cardiff, UK; 2 DECIPHer UKCRC Public Health Research Centre of Excellence, School of Social and Community Medicine, University of Bristol, Bristol, UK; 3 MRC Lifecourse Epidemiology Unit, University of Southampton, Southampton, UK; 4 Centre of Excellence in Intervention and Prevention Science, Melbourne, VIC Australia; 5 Department of Childhood, Families and Health, Institute of Education, University of London, London, UK; 6 Primary Care Unit, Department of Public Health and Primary Care, University of Cambridge, Cambridge, UK; 7 MRC/CSO Social and Public Health Sciences Unit, University of Glasgow, Glasgow, UK; 8 School of Health and Related Research, University of Sheffield, Sheffield, UK

## Abstract

Process evaluation is an essential part of designing and testing complex interventions. New MRC guidance provides a framework for conducting and reporting process evaluation studies

Attempts to tackle problems such as smoking and obesity increasingly use complex interventions. These are commonly defined as interventions that comprise multiple interacting components, although additional dimensions of complexity include the difficulty of their implementation and the number of organisational levels they target.^1^ Randomised controlled trials are regarded as the gold standard for establishing the effectiveness of interventions, when randomisation is feasible. However, effect sizes do not provide policy makers with information on how an intervention might be replicated in their specific context, or whether trial outcomes will be reproduced. Earlier MRC guidance for evaluating complex interventions focused on randomised trials, making no mention of process evaluation.^2^ Updated guidance recognised the value of process evaluation within trials, stating that it “can be used to assess fidelity and quality of implementation, clarify causal mechanisms and identify contextual factors associated with variation in outcomes.”^3^ However, it did not provide guidance for carrying out process evaluation.

**Summary points**

MRC guidance for developing and evaluating complex interventions recognised the importance of process evaluation within trials but did not provide guidance for its conduct
This article presents a framework for process evaluation, building on the three themes for process evaluation described in 2008 MRC guidance (implementation, mechanisms, and context)
It argues for a systematic approach to designing and conducting process evaluations, drawing on clear descriptions of intervention theory and identification of key process questions
While each process evaluation will be different, the guidance facilitates planning and conducting a process evaluation

## Developing guidance for process evaluation

In 2010, a workshop funded by the MRC Population Health Science Research Network discussed the need for guidance on process evaluation.^4^ There was consensus that researchers, funders, and reviewers would benefit from guidance. A group of researchers with experience and expertise in evaluating complex interventions was assembled to produce the guidance. In line with the principles followed in developing earlier MRC guidance documents, draft guidance was produced drawing on literature reviews, process evaluation case studies, workshops, and discussions at conferences and seminars. It was then circulated to academic, policy, and practice stakeholders for comment. Around 30 stakeholders provided written comments on the draft structure, while others commented during conference workshops run throughout the development process. A full draft was recirculated for further review, before being revised and approved by key MRC funding panels. 

Although the aim was to provide guidance on process evaluation of public health interventions, the guidance is highly relevant to complex intervention research in other domains, such as health services and education. The full guidance (www.populationhealthsciences.org/Process-Evaluation-Guidance.html) begins by setting out the need for process evaluation. It then presents a review of influential theories and frameworks which informed its development, before offering practical recommendations, and six detailed case studies. In this article, we provide an overview of the new framework and summarise our practical recommendations using one of the case studies as an example.

## MRC process evaluation framework

The new framework builds on the process evaluation themes described in the 2008 MRC complex interventions guidance (fig 1).^3^ Although the role of theory within evaluation is contested,^5^ ^6^ we concur with the position set out in the 2008 guidance, which argued that an understanding of the causal assumptions underpinning the intervention and use of evaluation to understand how interventions work in practice are vital in building an evidence base that informs policy and practice.^1^ Causal assumptions may be drawn from social science theory, although complex interventions will often also be informed by other factors such as past experience or common sense. An intervention as simple as a health information leaflet, for example, may reflect an assumption that increased knowledge of health consequences will trigger behavioural change. Explicitly stating causal assumptions about how the intervention will work can allow external scrutiny of its plausibility and help evaluators decide which aspects of the intervention or its context to prioritise for investigation. Our framework also emphasises the relations between implementation, mechanisms, and context. For example, implementation of a new intervention will be affected by its existing context, but a new intervention may also in turn change aspects of the context in which it is delivered.

**Figure fig1:**
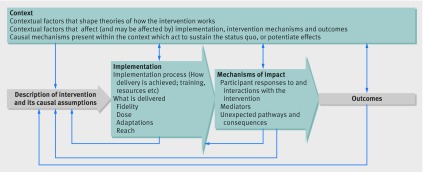
**Fig 1** Key functions of process evaluation and relations among them (blue boxes are the key components of a process evaluation. Investigation of these components is shaped by a clear intervention description and informs interpretation of outcomes)

### Implementation: what is implemented, and how?

An intervention may have limited effects either because of weaknesses in its design or because it is not properly implemented.^7^ On the other hand, positive outcomes can sometimes be achieved even when an intervention was not delivered fully as intended.^8^ Hence, to begin to enable conclusions about what works, process evaluation will usually aim to capture fidelity (whether the intervention was delivered as intended) and dose (the quantity of intervention implemented). Complex interventions usually undergo some tailoring when implemented in different contexts. Capturing what is delivered in practice, with close reference to the theory of the intervention, can enable evaluators to distinguish between adaptations to make the intervention fit different contexts and changes that undermine intervention fidelity.^9^ ^10^ Unresolved debates regarding adaption of interventions, and what is meant by intervention fidelity, are discussed at length in the full guidance.

In addition to what was delivered, process evaluation can usefully investigate how the intervention was delivered.^11^ ^12^ This can provide policy makers and practitioners with vital information about how the intervention might be replicated, as well as generalisable knowledge on how to implement complex interventions. Issues considered may include training and support, communication and management structures, and how these structures interact with implementers’ attitudes and circumstances to shape the intervention. 

Process evaluations also commonly investigate the “reach” of interventions (whether the intended audience comes into contact with the intervention, and how).^13^ There is no consensus on how best to divide the study of implementation into key subcomponents (such as fidelity, dose, and reach), and it is currently not possible to adjudicate between the various frameworks that attempt to do this. These issues are discussed further in the full guidance document.

### Mechanisms of impact: how does the delivered intervention produce change?

Exploring the mechanisms through which interventions bring about change is crucial to understanding both how the effects of the specific intervention occurred and how these effects might be replicated by similar future interventions.^14^ Process evaluations may test hypothesised causal pathways using quantitative data as well as using qualitative methods to better understand complex pathways or to identify unexpected mechanisms.^15^

### Context: how does context affect implementation and outcomes?

Context includes anything external to the intervention that may act as a barrier or facilitator to its implementation, or its effects. As described above, implementation will often vary from one context to another. However, an intervention may have different effects in different contexts even if its implementation does not vary.^16^ Complex interventions work by introducing mechanisms that are sufficiently suited to their context to produce change,^17^ while causes of problems targeted by interventions may differ from one context to another. Understanding context is therefore critical in interpreting the findings of a specific evaluation and generalising beyond it. Even where an intervention itself is relatively simple, its interaction with its context may still be highly complex.

## Functions of process evaluation at different stages of development, evaluation, and implementation 

The focus of process evaluation will vary according to the stage at which it is conducted. The MRC framework recommends a feasibility and piloting phase after an intervention has been developed.^1^ ^3^ At this stage, process evaluation can have a vital role in understanding the feasibility of the intervention and optimising its design and evaluation. However, at the next stage, evaluating effectiveness, the emphasis of process evaluation shifts towards providing greater confidence in conclusions about effectiveness by assessing the quantity and quality of what was delivered, and assessing the generalisability of its effectiveness by understanding the role of context. Even when a process evaluation has been conducted at the feasibility stage, another will usually be needed alongside the full trial because new problems are likely to emerge when the intervention is tested in a larger more diverse sample.

## Planning, designing, conducting, and reporting a process evaluation

Box 1 summarises the key recommendations of the new MRC guidance for process evaluation. Given the diversity of complex interventions, the aims and methods of process evaluations will vary, but there are common considerations when developing and planning any such evaluation. The recommendations are not intended to be prescriptive but to help researchers to make decisions. Throughout this section, we have illustrated our points using one of the six case studies included in the full guidance, the process evaluation of the Welsh national exercise referral scheme (NERS)^8^ ^18^ ^19^; this scheme aimed to improve physical activity through primary care referral to exercise professionals in local authority leisure centres.

**Box 1: Key recommendations for process evaluation**

**Planning**

- Carefully define the parameters of relationships with intervention developers or implementers
  Balance the need for sufficiently good working relationships to allow close observation, against the need to remain credible as independent evaluators
  Agree whether evaluators will take an active role in communicating findings as they emerge (and helping correct implementation challenges) or have a more passive role
- Ensure that the research team has the correct expertise. This may require:
  Expertise in qualitative and quantitative research methods
  Appropriate interdisciplinary theoretical expertise
- Decide the degree of separation or integration between process and outcome evaluation teams
  Ensure effective oversight by a principal investigator who values all evaluation components
  Develop good communication systems to minimise duplication and conflict between process and outcomes evaluations
  Ensure that plans for integration of process and outcome data are agreed from the outset

**Design and conduct**

- Clearly describe the intervention and clarify causal assumptions (in relation to how it will be implemented, and the mechanisms through which it will produce change, in a specific context)
- Identify key uncertainties and systematically select the most important questions to address
  Identify potential questions by considering the assumptions represented by the intervention
  Agree scientific and policy priority questions by considering the evidence for intervention assumptions and consulting the evaluation team and policy or practice stakeholders
  Identify previous process evaluations of similar interventions and consider whether it is appropriate to replicate aspects of them and build on their findings
- Select a combination of methods appropriate to the research questions:
  Use quantitative methods to measure key process variables and allow testing of pre-hypothesised mechanisms of impact and contextual moderators
  Use qualitative methods to capture emerging changes in implementation, experiences of the intervention and unanticipated or complex causal pathways, and to generate new theory
  Balance collection of data on key process variables from all sites or participants with detailed data from smaller, purposively selected samples
  Consider data collection at multiple time points to capture changes to the intervention over time

**Analysis**

- Provide descriptive quantitative information on fidelity, dose, and reach
- Consider more detailed modelling of variations between participants or sites in terms of factors such as fidelity or reach (eg, are there socioeconomic biases in who received the intervention?)
- Integrate quantitative process data into outcomes datasets to examine whether effects differ by implementation or prespecified contextual moderators, and test hypothesised mediators
- Collect and analyse qualitative data iteratively so that themes that emerge in early interviews can be explored in later ones
- Ensure that quantitative and qualitative analyses build upon one another (eg, qualitative data used to explain quantitative findings or quantitative data used to test hypotheses generated by qualitative data)
- Where possible, initially analyse and report process data before trial outcomes are known to avoid biased interpretation
- Transparently report whether process data are being used to generate hypotheses (analysis blind to trial outcomes), or for post-hoc explanation (analysis after trial outcomes are known)

**Reporting**

- Identify existing reporting guidance specific to the methods adopted
- Report the logic model or intervention theory and clarify how it was used to guide selection of research questions and methods
- Disseminate findings to policy and practice stakeholders
- If multiple journal articles are published from the same process evaluation ensure that each article makes clear its context within the evaluation as a whole:
  Publish a full report comprising all evaluation components or a protocol paper describing the whole evaluation, to which reference should be made in all articles
  Emphasise contributions to intervention theory or methods development to enhance interest to a readership beyond the specific intervention in question

### Planning a process evaluation

#### Working with intervention developers and implementers

High quality process evaluation requires good working relationships with all stakeholders involved in intervention development or implementation. These can be difficult to establish—for example, because these stakeholders have professional or personal interests in portraying the intervention positively, or see evaluation as threatening. However, without good relationships, close observation of the intervention can be challenging. Evaluators also need to ensure that they maintain sufficient independence to observe the work of stakeholders critically. The NERS process evaluation identified serious problems with the implementation of some intervention components.^19^ Evaluators needed to be close enough to the intervention to record these problems and understand why they occurred, yet sufficiently independent to report them to intervention stakeholders honestly. Transparent reporting of relationships with policy and practice stakeholders, and being mindful of how these affect the evaluation, is crucial.

One key challenge in working with intervention stakeholders is whether to communicate emerging findings. That is, should evaluators act as passive observers who feed findings back at the end of an evaluation or help to correct problems in implementation as and when they appear.^20^ A more active role is appropriate at the feasibility testing stage. However, when evaluating effectiveness, researchers will ideally not engage in continuous quality improvement activities because these may compromise the external validity of the evaluation. Agreeing systems for communicating information to stakeholders at the outset of the study may help to avoid perceptions of undue interference or that the evaluator withheld important information.

#### Resources and staffing

When planning a process evaluation, evaluators need to ensure that there is sufficient expertise and experience to decide on, and achieve, its aims. A process evaluation team will often require expertise in quantitative and qualitative research methods. Process evaluations will often need to draw on expertise from a range of relevant disciplines including, for example, public health, primary care, epidemiology, sociology, and psychology. Sufficient resources are required to allow collection and analysis of large quantities of diverse data, bearing in mind that analysis of qualitative data is especially time consuming.

#### Relationships within evaluation teams

Process evaluation will typically form part of a study that includes evaluation of outcomes and possibly cost effectiveness. Some evaluators choose to separate process and outcome teams, while in other cases they are combined. Box 2 gives some pros and cons of each model. If the teams are separate effective communications are necessary to prevent duplication or conflict; with combined teams, there is a need for transparency about how this might influence the conduct and interpretation of the evaluation. Effective integration of evaluation components is more likely when members of a team respect and value each other’s work, and when the overall study is overseen by a principal investigator who values integration.^21^

**Box 2: Separation or integration of process evaluation and outcome evaluation teams?**

**Arguments for separation**

- Separation may reduce potential biases in analysis of outcomes data arising from feedback on the perceived functioning of the intervention
- In controlled trials, process evaluators cannot be blinded to treatment condition. Those collecting or analysing outcomes data ought to be blinded where possible
- Analysing process data without knowledge of trial outcomes prevents fishing for explanations and biasing interpretations. Although it may not always be practical to delay outcomes analysis until process analyses are complete, if separate researchers are responsible for each part it may be possible to conducted the analyses concurrently without biasing the results
- Process evaluation may produce data that would be hard for those with vested interests in the trial to analyse and report dispassionately
- If implementers or participants have concerns about a trial, a degree of separation from the trial may make it easier for process evaluators to build rapport and understand their concerns

**Arguments for integration**

- Process evaluators and outcomes evaluators will want to work together to ensure that data on implementation can be integrated into analysis of outcomes, or that data on emerging process issues can be integrated into trial data collections
- Data on intermediate outcomes and causal processes identified by process evaluators may inform integration of new measures into outcomes data collections
- If some relevant process measures are already being collected as part of the outcomes evaluation, it is important to avoid duplication of efforts and reduce measurement burden for participants
- One component of data collection should not compromise another. For example, if collection of process data is causing a high measurement burden for participants, this may lead to lower response to outcomes assessments

### Designing and conducting a process evaluation

#### Describing the intervention and clarifying causal assumptions

A clear description of the intended intervention, how it will be implemented, and how it is expected to work, will ideally have been developed before evaluation. In such cases, designing a process evaluation will begin by reviewing these descriptions to decide what requires investigation. Any ambiguity over what the intervention is, or how it is intended to work, should be resolved with the intervention developers before the design of the process evaluation is finalised. Evaluators of NERS had limited involvement in the development of the intervention, which was a Welsh government policy initiative. Hence, when evaluation began, some ambiguity remained over the content of the intervention and how it was intended to work. Evaluators worked with intervention developers to resolve this ambiguity, but as this took place after the evaluation had started, the time available to develop robust measures of some key activities was limited.^8^

It is useful if interventions and their evaluations draw explicitly on existing theories so that these can be tested and refined. However, when an intervention’s development is driven by other factors, such as experience or common sense, it is important to be open about this and clear about what these assumptions are, rather than trying to force an established theoretical framework to fit the intervention. Evaluators should also avoid focusing narrowly on inappropriate theories from a single discipline. For example, psychological theory may be useful for interventions that work at the individual level but is less useful when intervening with organisations or at wider social levels.^22^

Depicting the intervention in a logic model can help clarify causal assumptions.^23^ Fig 2 gives an example for INCLUSIVE, a school based intervention that aimed to reduce bullying and improve student health by implementing “restorative practices” across the whole school.^24^ The logic model was based on Markham and Aveyard’s theory of human functioning and school organisation, which suggests that health benefits would be mediated by whether students were connected to their school’s learning and community.^25^ This led the authors to identify measures of commitment and belonging as intermediate outcomes.^26^

**Figure fig2:**
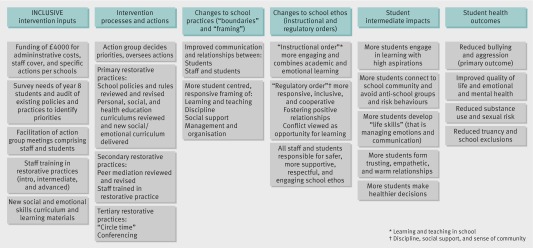
**Fig 2** Logic model for the INCLUSIVE intervention to reduce violence and aggression in schools^24^

#### Learning from previous process evaluations

When designing a process evaluation, it is important to be mindful that the results may later be included in systematic reviews. Process evaluation will provide the information on implementation and context that Waters and colleagues argue is essential if reviews are to assist decision makers.^27^ It is therefore helpful if process evaluations of similar interventions build on one another’s findings, using comparable methods if possible, so that reviewers can make meaningful comparisons across studies.

#### Deciding core research questions

Process evaluations cannot expect to provide answers to all of the uncertainties of a complex intervention.^28^ It is generally better to answer the most important questions well than to try to answer too many questions and do so unsatisfactorily. To identify core questions, evaluators may start by listing causal assumptions within the intervention manual or logic model and establishing which have the most limited evidence base. This can be done by reviewing the literature, consultation with policy and practice stakeholders, and discussions within the research team. Complex interventions are inherently unpredictable. Evaluators may therefore identify additional questions during the course of their evaluation. Hence, although clear focus from the outset is vital, process evaluations must be designed with sufficient flexibility and resources to allow important emerging questions to be addressed.

#### Selecting methods

Figure 3 lists some common data collection and analysis methods adopted by process evaluations, the merits of which should be considered carefully in relation to the research questions. Process evaluation of complex interventions usually requires a combination of quantitative and qualitative methods, but their relative importance may vary according to the status of the evidence base or stage of the evaluation process. At the feasibility and piloting stage, basic quantitative measures of implementation may be combined with in-depth qualitative data to provide detailed understandings of intervention functioning on a small scale. 

**Figure fig3:**
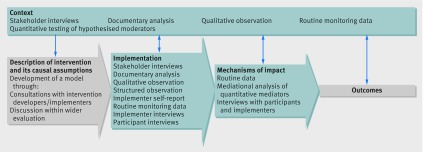
**Fig 3** Commonly used data collection and analysis methods for process evaluation

When evaluating effectiveness, collection of quantitative process measures to allow testing of hypothesised pathways or to measure contextual factors may be a priority. If directly relevant qualitative data are already available (for example, from an earlier feasibility study), evaluators may choose not to collect extensive qualitative process data while evaluating effectiveness. However, collecting additional qualitative data may still help in understanding issues arising from the movement from a small scale feasibility study to a larger scale evaluation involving greater diversity in implementers, settings, and participants.

Key methodological considerations include sampling and timing of data collection. Interviewing every implementer may not provide greater insights than interviewing a small well selected sample, and may lead to overwhelming volumes of data. Conducting observations in every site may be prohibitively expensive and unduly influence implementation. Conversely, there are dangers in collecting data from only a few sites in order to draw conclusions regarding the intervention as a whole.^28^ Hence, when feasible, it is often useful to combine quantitative data on key process variables from all sites or participants with in-depth qualitative data from samples purposively selected along dimensions expected to influence the functioning of the intervention. Collecting data at multiple time points may be useful because interventions can suffer from teething problems which are rectified as the evaluation progresses.

Within the NERS process evaluation, quantitative measures included structured observations of audio recorded patient consultations. These were used to examine aspects of fidelity (such as consistency with motivational interviewing principles), and dose (such as the duration of consultations). Sociodemographic patterning in entry to the scheme (reach) was evaluated using routinely collected monitoring data.^8^ Quantitative measures of hypothesised psychological mechanisms, including motivation for exercise and confidence, were collected as part of the trial.^18^ Qualitative interviews were conducted with patients, exercise professionals, scheme coordinators, and health professionals. These focused on challenges in implementation across contexts and how NERS was perceived to work in practice.^8^

### Analysis of process data, and integration of process and outcome data

Analysis of quantitative process data will usually begin with descriptive statistics relating to questions such as fidelity, dose, and reach. Subsequently, integrating quantitative process measures into outcomes datasets can help to understand how, for example, implementation variability affected outcomes (on-treatment analyses) and test hypotheses arising from qualitative analyses. Some argue that initial analysis of process data should be conducted before the outcomes analysis to avoid biased interpretation of process data.^29^ If this model is followed, process data may provide prospective insights into why evaluators might subsequently expect to see positive or negative overall effects and generate hypotheses about how variability in outcomes may emerge.^30^

In the NERS process evaluation, implementation measures indicated that the intervention comprised a common core of health professional referrals to discounted, supervised, group based exercise. However, some activities, such as motivational interviewing and goal setting, were poorly delivered.^8^ Nevertheless, qualitative data (analysed before trial outcomes were available) indicated that patient motivation was supported by other mechanisms, such as social support from other patients.^8^ Subsequently, integration of quantitative measures of psychological change mechanisms with trial outcomes data indicated that significant improvement in physical activity was explained by change in motivation for exercise.^18^ Hence, the integration of qualitative and quantitative process data with trial outcomes helped to clarify complex causal pathways.

### Reporting findings 

Reporting guidelines for health research are available on the EQUATOR network website (www.equator-network.org/home), but such guidelines for process evaluations are challenging because they vary so much. Key considerations include reporting relations between quantitative and qualitative components, and the relation of the process evaluation to other evaluation components, such as outcomes or economic evaluation. It is also useful to report assumptions about how the intervention works (ideally in a logic model), and how these informed the selection of research questions and methods.^31^ Reporting in the peer reviewed literature will often require multiple articles. To maintain sight of the broader picture, all journal articles should refer to other articles published from the study or to a protocol paper or report that clarifies how the component publications relate to the overall evaluation. When process evaluation has been conducted to interpret trial outcomes, interpretation needs to be clear in the published papers, with process evaluation data linked, in discussion, to trial outcomes. It is also important to report in lay formats for people who delivered the intervention or who will be making decisions about its future implementation.

## Notes

Cite this as: *BMJ* 2015;350:h1258
